# The complete mitochondrial genome of Lesser Sand-Plover *Charadrius mongolus atrifrons* and its phylogenetic position

**DOI:** 10.1080/23802359.2021.1972482

**Published:** 2021-09-09

**Authors:** Shangmingyu Zhang, Xiaofeng Zheng, Chuang Zhou, Kunlin Yang, Yongjie Wu

**Affiliations:** a Key Laboratory of Bio‐resources and Eco‐environment, Ministry of Education, College of Life Sciences, Sichuan University, Chengdu, China; b Sichuan Key Laboratory of Conservation Biology on Endangered Wildlife, College of Life Sciences, Sichuan University, Chengdu, China; c Wildlife Resources Survey and Conservation Station of Sichuan Province, Chengdu, China

**Keywords:** *Charadrius mongolus atrifrons*, mitochondrial genome, phylogenetic analysis

## Abstract

The Lesser Sand-Plover (*Charadrius mongolus atrifrons*) is a small shorebird in Charadriiformes. Here we assembled the complete mitochondrial genome of *C. m. atrifrons* (Aves: Charadriiformes) which is 16,919 bp in length and consisting of 13 protein-coding (PCGs), 2 ribosomal RNA, 22 transfer RNA and 1 control region. The overall A + T content of was 55.5%. The Maximum Likelihood (ML) tree based on the 12 concatenated mitochondrial protein-coding genes (except ND6 gene) placed *C. m. atrifrons* in a clade with *C. alexandrines* but separate from *C. vociferus*.

Charadriiformes is a species-rich clade of shorebirds, which is currently represented by 13 family-level taxa in China (Zheng 2017). The phylogenetic relationships within shorebirds have been well studied based on nuclear and/or short mitochondrial sequences (Barth et al. 2013; Remedios et al. 2015; Chen et al. 2018), but the phylogenetic placement of many species has not been addressed with complete mitochondrial genomes. To date, correctly identified complete mitochondrial genomes have been published for only six species of Charadriidae, which hampers studies of the ecology, evolutionary biology and population genetics of these shorebirds. The Lesser Sand-Plover (*Charadrius mongolus atrifrons*) is a long-distance migratory bird in Charadriiformes, whose mitochondrial DNA have not been well-studied. In this study, we sequenced and analyzed the complete mitochondrial genome of *C. m. atrifrons*, providing a basis for future studies of population genetics, evolution, phylogeny and conservation genetics.

Muscle tissue of a wild *C. m. atrifrons* collected from an airport protection facility was gathered from Nagri Kunsha Airport (80.055442E, 32.100026 N) in July 2020. The specimen was stored in the Natural Museum of Sichuan University with a voucher number of QZKK063 (Curator name: Jianghong Ran; Email: rjhong-01@163.com). The total genomic DNA was extracted using the M5 HiPer Universal DNA Mini Kit following the manufacturer’s instructions. The mitogenome of *C. m. atrifrons* were generated by amplification of overlapping Polymerase Chain Reaction (PCR) fragments. The thirteen fragments were amplified using the universal primers (Amer et al. 2013) following the instructions of 2× Rapid Taq Master Mix (Vazyme Biotech Co., Ltd). Sequences obtained were aligned and annotated using the software SeqMan (DNAStar, Inc.) and MITOS Web Server (Bernt et al. 2013), respectively.

The circular mitochondrial genome of *C. m. atrifrons* (GenBank accession number MW298528) was 16,919 bp in length, consisting of 13 protein-coding (PCGs), 2 ribosomal RNA (rRNA), 22 transfer RNA (tRNA) and 1 control region, which is consistent with other vertebrate mitogenomes (Xie et al. 2016). All the genes of *C. m. atrifrons* are encoded on the H-strand with the exception of ND6 and 8 tRNAs (tRNA^Gln^, tRNA^Ala^, tRNA^Asn^, tRNA^Cys^, tRNA^Tyr^, tRNA^Ser(UCN)^, tRNA^Pro^ and tRNA^Glu^). The overall nucleotide base composition was 32.0% A, 30.5% C, 13.9% G, and 23.5% T. The overall A + T content of 55.5%. The AT skew was calculated as 0.15.

To determine the phylogenetic position of *C. m. atrifrons*, PhyloSuite (Zhang et al. 2020) was used to construct a maximum-likelihood (ML) phylogenetic tree of seven species (*Pluvialis apricaria*, *P. fulva*, *Vanellus cinereus*, *V. vanellus Charadrius vociferus, C. alexandrinus,* and *C. m. atrifrons*). *Tringa nebularia* (Zhang et al. 2019) was used as an outgroup (Figure 1). GTR + F + I + G4 was selected as the substitution model according to the Bayesian Information Criterion (BIC) test based on Modeltest. The Maximum Likelihood phylogeny placed the members of Charadriidae in four major groups. *C. m. atrifrons* was placed with *C. alexandrinus* but distant from *C. vociferus*. This pattern is consistent with the results of Barth et al. (2013) and Remedios et al. (2015) who found that the genus *Charadrius* is paraphyletic due to the inclusion of *Vanellus*. Indeed, it has been suggested that some species of *Charadrius*, including *C. m. atrifrons*, should be placed in a different genus *Anarhynchus* (Sangster et al. 2016). This pattern was inconsistent with the placement of *Charadrius* plovers and *Vanellus* lapwings in separate genera (Dickinson & Remsen, 2013). In conclusion, our study described the complete mitogenome of *C. m. atrifrons* and investigated its phylogenetic position, which may benefit future studies of ecology, evolutionary biology and population genetics in shorebirds.

**Figure 1.**
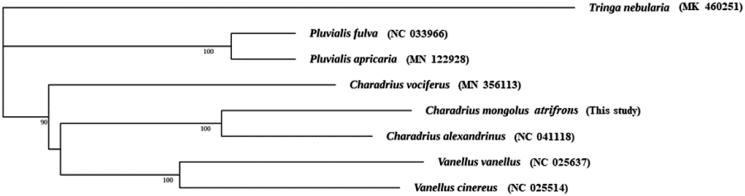
Phylogenetic tree of *Charadrius mongolus atrifrons* based on the maximum likelihood (ML) analysis of 12 concatenated mitochondrial protein-coding genes. *Tringa nebularia* was set as outgroup. The bootstrap values are based on 1000 replicates and shown at the nodes.

## Data Availability

The genome sequence data that support the findings of this study are openly available in GenBank of NCBI at https://www.ncbi.nlm.nih.gov, under the accession MW298528. The associated BioProject, SRA, and Bio-Sample numbers are PRJNA741831, SRP326305 and SAMN19908120 respectively.

## References

[CIT0001] Amer S, Ahmed MM, Shobrak M. 2013. Efficient newly designed primers for the amplification and sequencing of bird mitochondrial genomes. Biosci Biotechnol Biochem. 77(3):577–581. 23470741 10.1271/bbb.120819

[CIT0002] Barth JMI, Matschiner M, Robertson BC. 2013. Phylogenetic position and subspecies divergence of the endangered New Zealand Dotterel (*Charadrius obscurus*). PLoS One. 8(10):e78068. 24205094 10.1371/journal.pone.0078068 PMC3808304

[CIT3003] Bernt M, Donath A, Juhling F, Externbrink F, Florentz C, Fritzsch G, Putz J, Middendorf M, Stadler P F. 2013. MITOS: improved de novo metazoan mitochondrial genome annotation. Mol Phylogenet Evol. 69(2):313–319. 10.1016/j.ympev.2012.08.023 22982435

[CIT0003] Chen W, Zhang C, Pan T, Liu W, Li K, Hu C, Chang Q. 2018. The mitochondrial genome of the Kentish Plover *Charadrius alexandrinus* (Charadriiformes: Charadriidae) and phylogenetic analysis of Charadrii. Genes Genomics. 40(9):955–963. 30155708 10.1007/s13258-018-0703-3

[CIT0004] Dickinson EC, Remsen JV. 2013. The Howard and Moore complete checklist of the birds of the world. 4th ed, Vol. 1. Non-passerines. London: Aves Press.

[CIT0005] Remedios ND, Lee P, Burke T, Székely T, Küpper C. 2015. North or South? phylogenetic and biogeographic origins of a globally distributed avian clade. Mol Phylogenet Evol. 89:151–159. 25916188 10.1016/j.ympev.2015.04.010

[CIT0006] Sangster G, Collinson JM, Crochet P-A, Kirwan GM, Knox AG, Parkin DT, Votier SC. 2016. Taxonomic recommendations for Western Palearctic birds: 11th Report. Ibis. 158(1):206–212.

[CIT0007] Xie W, Hu C, Yu T, Yang R, Chang Q. 2016. The complete mitochondrial genome of *Vanellus cinereus* (Charadriiformes: Charadriidae). Mitochondrial DNA A DNA Mapp Seq Anal. 27(3):1726–1727. 25242177 10.3109/19401736.2014.961141

[CIT0008] Zhang D, Gao F, Jakovlić I, Zou H, Zhang J, Li WX, Wang GT. 2020. Phylosuite: an integrated and scalable desktop platform for streamlined molecular sequence data management and evolutionary phylogenetics studies. Mol Ecol Resour. 20(1):348–355. 31599058 10.1111/1755-0998.13096

[CIT0009] Zhang F, Zhang N, Huang Z, Xiang Q, Chen W. 2019. The complete mitochondrial genome of Common Greenshank Tringa nebularia. Mitochondrial DNA Part B. 4(1):1588–1589.

[CIT0010] Zheng GM. 2017. A checklist on the classification and distribution of birds in China. 3rd ed. Beijing: Science Press.

